# 8-Oxoguanine DNA Glycosylase 1 Upregulation as a Risk Factor for Obesity and Colorectal Cancer

**DOI:** 10.3390/ijms24065488

**Published:** 2023-03-13

**Authors:** Jesús Pilo, Libia Alejandra García-Flores, Mercedes Clemente-Postigo, Isabel Arranz-Salas, Julia Alcaide, Maria Ramos-Fernandez, José Lozano, Hatim Boughanem, Pallavi Kompella, Manuel Macías-González

**Affiliations:** 1 Department of Endocrinology and Nutrition, Virgen de la Victoria University Hospital, 29010 Malaga, Spain; 2 Institute of Biomedical Research in Malaga (IBIMA)-Bionand Platform, University of Malaga, 29590 Malaga, Spain; 3 Spanish Biomedical Research Center in Physiopathology of Obesity and Nutrition (CIBERObn), Instituto de Salud Carlos III, 28029 Madrid, Spain; 4 Department of Cell Biology, Genetics, and Physiology, Faculty of Science, University of Malaga, 29590 Malaga, Spain; 5 Department of Cell Biology, Physiology and Immunology, Maimónides Biomedical Research Institute of Córdoba (IMIBIC), Reina Sofia University Hospital, University of Córdoba, 14004 Córdoba, Spain; 6 Division of Anatomical Pathology, Hospital Universitario Virgen de la Victoria, 29010 Malaga, Spain; 7 Department of Human Physiology, Human Histology, Anatomical Pathology and Physical Education, University of Malaga, 29010 Malaga, Spain; 8 Medical Oncology Intercenter Unit, Regional and Virgen de la Victoria University Hospitals, IBIMA, 29010 Málaga, Spain; 9 Unidad de Gestion Clinica Cirugía General y del Aparato Digestivo, Virgen de la Victoria University, 29010 Malaga, Spain; 10 Department of Biochemistry and Molecular Biology, University of Malaga, 29010 Málaga, Spain; 11 Unidad de Gestión Clinica Medicina Interna, Lipids and Atherosclerosis Unit, Maimonides Institute for Biomedical Research in Córdoba, Reina Sofia University Hospital, University of Córdoba, 14004 Córdoba, Spain; 12 Division of Pharmacology and Toxicology, College of Pharmacy, The University of Texas at Austin, Austin, TX 78712, USA

**Keywords:** colorectal cancer, CRC, repair genes, OGG1, methylation, obesity, DNA repairs, inflammation

## Abstract

DNA damage has been extensively studied as a potentially helpful tool in assessing and preventing cancer, having been widely associated with the deregulation of DNA damage repair (DDR) genes and with an increased risk of cancer. Adipose tissue and tumoral cells engage in a reciprocal interaction to establish an inflammatory microenvironment that enhances cancer growth by modifying epigenetic and gene expression patterns. Here, we hypothesize that 8-oxoguanine DNA glycosylase 1 (OGG1)—a DNA repair enzyme—may represent an attractive target that connects colorectal cancer (CRC) and obesity. In order to understand the mechanisms underlying the development of CRC and obesity, the expression and methylation of DDR genes were analyzed in visceral adipose tissue from CRC and healthy participants. Gene expression analysis revealed an upregulation of *OGG1* expression in CRC participants (*p* < 0.005) and a downregulation of *OGG1* in normal-weight healthy patients (*p* < 0.05). Interestingly, the methylation analysis showed the hypermethylation of *OGG1* in CRC patients (*p* < 0.05). Moreover, expression patterns of *OGG1* were found to be regulated by vitamin D and inflammatory genes. In general, our results showed evidence that *OGG1* can regulate CRC risk through obesity and may act as a biomarker for CRC.

## 1. Introduction

According to the last Global Cancer Statistics 2020, colorectal cancer (CRC) is the third most frequent cancer [1] and the second most common cause of cancer-related death worldwide (9.4% of all cancer deaths) [2]. Epidemiological data suggest that a 30–70% increased risk of CRC can be attributed to obesity [3]. Increases in visceral adipose tissue (VAT) are linked to adipose tissue dysfunction and can induce chronic local and systemic low-grade inflammation. This inflammation is due to an increase in the production of pro-inflammatory cytokines such as interleukin 6 (IL6) and tumor necrosis factor-α (TNF-α), as well as the production of reactive oxygen species (ROS) as a result of oxidative stress [4]. Furthermore, increased levels of oxidative stress caused by ROS may cause DNA damage—a well-recognized risk factor for colorectal oncogenesis [5].

ROS can damage DNA in different pathways. However, 8-oxoguanine (8-oxoG) stands out as one of the most common DNA lesions observed as a result of exposure to ROS products [6]. 8-OxoG is a tautomer that can result in a mismatched pairing with adenine [7]. To eliminate 8-oxoG, all organisms have developed several DNA repair strategies [8]. 8-Oxoguanine DNA glycosylase (encoded by the *OGG1* gene on chromosome 3p25) is a key enzyme in correcting this mismatch. This multifunctional protein repairs the guanine lesions in mammalian cells through stepwise base excision repair (BER) [9,10,11]. Accordingly, several studies have shown controversial results on the transcriptional profile of the *OGG1* gene in cancer tissue [10,11,12]. Furthermore, multiple studies have evaluated the association between *OGG1* polymorphisms and the risk of CRC [13,14,15,16]. However, the results are still conflicting [17]. Hence, estimating the DNA damage or disruptions in DNA repair could potentially be helpful in the risk assessment and prevention of obesity-associated metabolic disorders and cancers [18,19]. However, the causative aspects underpinning this association are only partially understood [20,21].

Therefore, we hypothesize that obesity could alter the expression and methylation profile of the *OGG1* gene in VAT in the context of cancer, ultimately contributing to DNA damage and increasing the risk of CRC. Then, our study aims to examine the gene expression and methylation of *OGG1* in the adipose tissue of patients with CRC. In addition, we intend to evaluate the Ser326Cys *OGG1* polymorphism to understand its association with obesity and cancer. Finally, we investigate an in vitro model in adipocytes to investigate the role of vitamin D in DNA repair.

## 2. Results

### 2.1. Baseline and General Characteristics of Participants

Table 1 presents the anthropometric and biochemical variables of healthy participants and patients with CRC. As observed, the two groups had significant differences in anthropometric variables, such as body mass index (BMI). Patients with CRC had a lower BMI than healthy participants (*p* = 0.001). Furthermore, there were significant differences in biochemical variables related to glucose and lipid metabolism. Accordingly, patients with CRC had increased glucose levels compared to healthy participants (*p* < 0.001). Conversely, total cholesterol and LDL were higher in healthy participants in comparison with patients with CRC (*p* < 0.001). Furthermore, CRC patients also had lower triglycerides and HDL than healthy participants (*p* < 0.001). Finally, serum levels of 25-hydroxyvitamin D (25(OH)D) were lower in patients with CRC when compared to healthy participants (*p* < 0.001). Supplementary Table S1 shows the differences between healthy participants and patients with CRC, grouped by BMI.

The frequencies of the *OGG1* Ser302Cys polymorphism in all subjects are summarized in Table 1 and Supplementary Table S1. Non-significant differences were found among the genotypes and allele frequencies between the healthy participants and patients with CCR. The Ser302Cys allele and genotype distributions deviated from the Hardy–Weinberg equilibrium in all participants (*p* = 0.023*) and in patients with CRC (*p* = 0.006 *), but not in healthy participants (*p* = 0.45). The minor allele frequencies of the *OGG1* Ser302Cys polymorphism were 0.07 for all participants, 0.05 for the healthy participants, and 0.11 for patients with CRC.

### 2.2. Transcriptional and Methylation Profiles of the OGG1 Gene in Obesity and Colorectal Cancer

**Figure 1:**
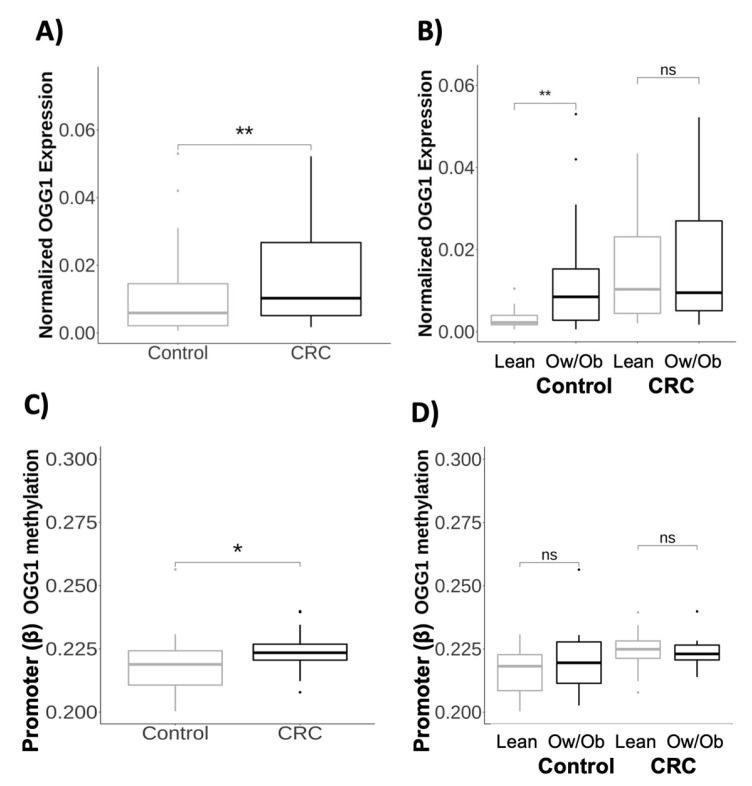
Gene expression and methylation profile of *OGG1* in visceral adipose tissue from patients with obesity and colorectal cancer: (**A**) Normalized gene expression of *OGG1* comparing healthy participants (N = 54) and patients with CRC (N = 65). (**B**) Normalized gene expression of *OGG1* comparing healthy lean participants (N = 13) with overweight/obese participants (N = 41). In addition, we compared lean (N = 22) and overweight/obese patients with CRC (N = 43). (**C**) Promoter methylation of *OGG1* comparing healthy participants (N = 24) and patients with CRC (N = 23). The promoter contained the following CpG sites: cg11841349, cg14201528, cg15357639, cg17285536, cg17319894, cg19391888, cg25415932, and cg05439191. (**D**) Promoter methylation of *OGG1* comparing healthy lean participants (N = 7) with overweight/obese participants (N = 17). In addition, we compared lean (N = 11) and overweight/obese patients with CRC (N = 12). Gene expression was normalized using the *PPIA* gene and the formula: 2^−ΔCt^. Abbreviations: CRC: colorectal cancer; OGG1: OGG1 8-oxoguanine DNA glycosylase 1; Ow/Ob: overweight/obese; ns: non-significant. Asterisks indicate significant values according to the test (* *p* < 0.05; ** *p* < 0.01).

We evaluated the transcriptional and epigenetic profiles in obesity and CRC. As shown in Figure 1A, *OGG1* expression was upregulated in VAT patients with CRC compared to healthy participants (*p* < 0.01). We also found that *OGG1* was upregulated in whole blood in patients with CRC compared to healthy participants (*p* < 0.05) (Supplementary Figure S1A). Furthermore, we divided the participants by obesity grade, in which lean participants had a BMI < 25 Kg/m^2^, while participants with overweight/obesity (Ow/Ob) had a BMI ≥ 25 Kg/m^2^. Our analysis showed that healthy participants with Ow/Ob had higher *OGG1* expression than lean healthy participants (*p* < 0.01). Nevertheless, no significant differences were observed between lean and Ow/Ob patients with CRC (Figure 1B). The complete analysis is summarized in Supplementary Figure S1B.

Furthermore, we analyzed the whole promoter methylation of the *OGG1* gene. Our analysis revealed that the promoter methylation of the *OGG1* gene was higher in patients with CRC in comparison with healthy participants (*p* < 0.05) (Figure 1C). However, non-significant differences were observed when we compared healthy participants with Ow/Ob and lean healthy participants and lean and Ow/Ob patients with CRC (Figure 1D). The complete analysis is summarized in Supplementary Figure S1C. A Pearson’s correlation test was conducted to understand the relationship between *OGG1* methylation and expression. We found a significant negative correlation between *OGG1* methylation in body gene and *OGG1* expression (r = −0.354, *p* = 0.039) (Supplementary Figure S1D). Surprisingly, no correlation was found between the promoter *OGG1* methylation and *OGG1* expression.

### 2.3. Association between OGG1 and Metabolic/DNA Repair Genes

**Figure 2:**
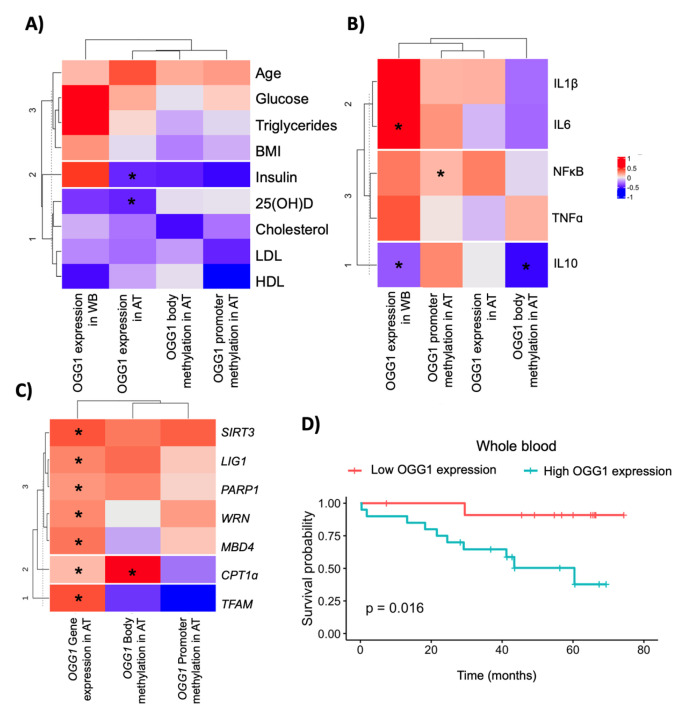
*OGG1* expression in adipose tissue and metabolic and DNA repair genes: (**A**) Heatmap of Pearson’s correlation between *OGG1* expression and methylation, and anthropometric and biochemical variables in all participants. (**B**) Heatmap of Pearson’s correlation between *OGG1* expression and methylation and gene expression of inflammatory genes in visceral adipose tissue in all participants. (**C**) Heatmap of Pearson’s correlation between *OGG1* expression and methylation and gene expression of DNA repair genes in visceral adipose tissue from healthy participants (**D**) Kaplan–Meier estimates for overall survival according to gene expression of *OGG1* (low vs. high under the median value) in whole blood. The asterisks indicate a significant correlation between variables according to Pearson’s correlation test (* *p* < 0.05). Abbreviations: AT: adipose tissue; WB: whole blood.

Pearson’s analysis was conducted to evaluate the relationships between *OGG1* and biochemical variables. We observed that *OGG1* expression in VAT was negatively associated with 25(OH)D (r = −0.02; *p* = 0.03) and insulin levels (r = −0.02; *p* = 0.03) (Figure 2A). As for the inflammatory genes, *OGG1* expression in VAT was associated with NF-κB expression (r = 0.29, *p* = 0.003). *OGG1* expression in whole blood was negatively associated with IL10 (r = −0.34, *p* = 0.015) and positively associated with IL6 (r = 0.51, *p* = 0.047). Furthermore, *OGG1* body methylation was negatively associated with IL10 expression (r = −0.56; *p* = 0.008) (Figure 2B). Finally, *OGG1* expression measured in the adipose tissue from healthy participants was associated with the majority of DNA repair genes, including *SIRT3* (r = 0.81, *p* < 0.001), *LIG1* (r = 0.71, *p* < 0.001), *PARP1* (r = 0.56, *p* < 0.001), *WRN* (r = 0.79, *p* < 0.001), *MBD4* (r = 0.74, *p* < 0.001), *CPT1ɑ* (r = 0.47, *p* < 0.001), and *TFAM* expression (r = 0.63, *p* = 0.04). *OGG1* body methylation was associated with *CPT1ɑ* expression (r = 0.99; *p* = 0.031) (Figure 2C).

### 2.4. OGG1 as a Potential Candidate Biomarker in Colorectal Cancer Outcomes

A regression analysis was conducted to understand the relationship between *OGG1* in the adipose tissue and the risk of CRC (Table 2). After adjusting for age, sex, and BMI, *OGG1* expression in VAT was positively associated with an increased risk of CRC (β = 7.21 (3.09), *p* < 0.05), explaining up to 30% of the variability. The area under the curve of this model was 0.686 (0.586–0.787) (Supplementary Figure S1E). However, *OGG1* expression in whole blood, its promoter, and bodily methylation of the *OGG1* gene were not linked to an increased risk of CRC. In the Kaplan–Meier analysis for overall survival, *OGG1* expression in VAT (low vs. high under the median value) was not associated with overall survival (Supplementary Figure S1F) (*p* = 0.33). However, overall survival was associated with *OGG1* expression in whole blood (low vs. high under the median value). High *OGG1* expression was associated with worse survival compared to low *OGG1* expression (*p* = 0.016) (Figure 2D). We also found that *OGG1* expression in adipose tissue was lower in late-stage cancer when compared with patients with early-stage cancer (*p* = 0.028) (Supplementary Figure S1G).

As for the mutational analysis, we evaluated the association between the *OGG1* Ser302Cys polymorphism and serum biomarkers for oxidative stress, such as Hb1Ac, transferrin, ferritin, and alkaline phosphatase (ALP). We did not observe significant differences between the CC (dominant group) and CG/GG (recessive group) groups (Supplementary Figure S2A,B). However, we found that the CC group had a trend of increased ferritin values compared to the CG/GG group (*p* = 0.066) (Supplementary Figure S2C). In addition, we found that the CC group had a higher value of ALP when compared to the CG/GG group (*p* = 0.029) (Supplementary Figure S2D).

### 2.5. In Vitro Validation of the OGG1 Gene Profile in the Adipose Tissue and Adipocytes

**Figure 3:**
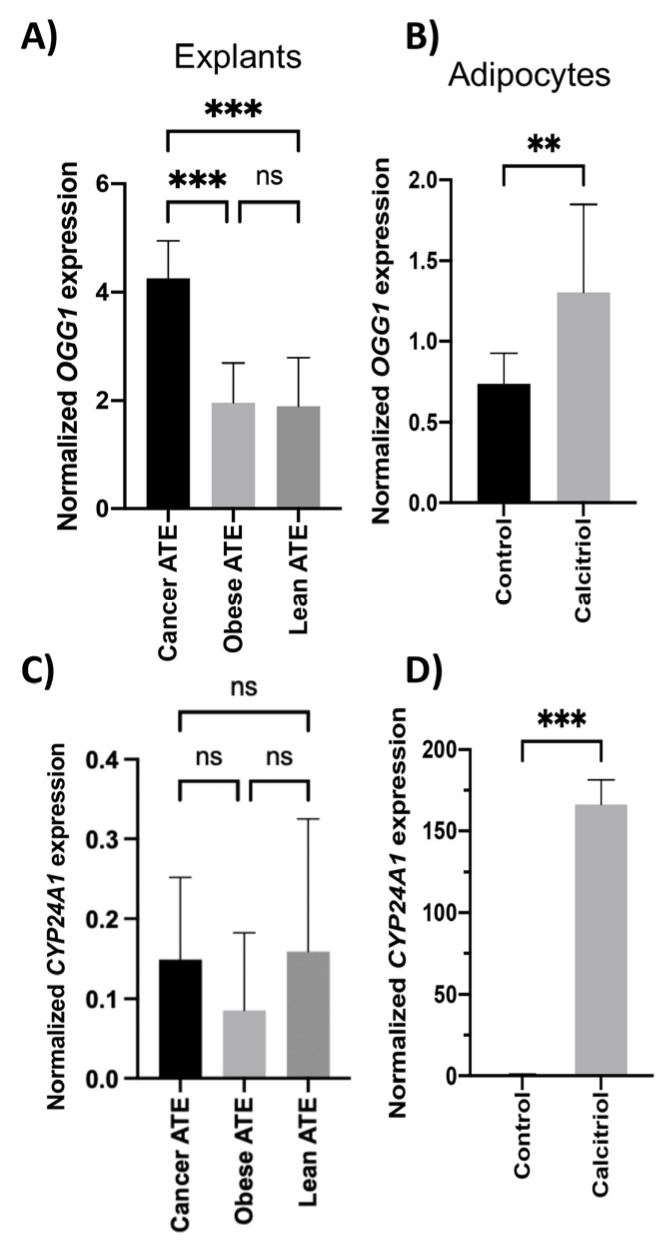
In vitro validation of the *OGG1* gene profile in the adipose tissue and adipocytes: Total RNA from explants was extracted, and gene expression was measured. Normalized gene expression of (**A**) *OGG1* and (**C**) *CYP24A1* was calculated by comparing explants from healthy lean participants (N = 3), healthy obese participants (N = 3), and patients with CRC (N = 4). Adipocytes were treated with 0.5 µM of calcitriol for 24 h. (**B**) *OGG1* and (**D**) *CYP24A1* was calculated by comparing control and treated adipocytes. The asterisks indicate significant values according to the test (** *p* < 0.01; *** *p* < 0.001). Gene expression was normalized using the *PPIA* gene and the formula 2^−ΔCt^. ns: no significant.

To understand the biological significance of previous findings, we evaluated patient-derived explants from lean and obese participants and those from patients with CRC. We observed that adipose tissue explants from patients with CRC showed higher expression of *OGG1* compared to explants from lean (*p* < 0.001) and obese healthy participants (*p* < 0.001) (Figure 3).

Therefore, we treated the explants with 1 µM of calcitriol. However, we did not observe an effect on the *OGG1* expression of the treated explants compared to the controls (Supplementary Figure S2E). Nevertheless, adipocytes treated with 0.5 µM of calcitriol showed increased *OGG1* expression when compared to the controls (*p* < 0.01) (Figure 3B). Furthermore, we tested the treatment efficacy with calcitriol and measured the gene expression of *CYP24A1*. This is because *CYP24A1* is a target gene of calcitriol, which is mediated by the VDR, which is a transcription factor that binds to specific DNA sequences in the promoter region of the *CYP24A1* gene [22]. In the explants treated with calcitriol, we did not observe significant differences between explants from lean, obese, and patients with CRC (Figure 3C). Furthermore, calcitriol treatment significantly increased the expression between control and treated explants in lean participants, obese patients, and CRC patients (*p* < 0.01, *p* < 0.01, and *p* < 0.001, respectively) (Supplementary Figure S2F). As for the adipocytes treated with calcitriol, we observed that treatment with calcitriol significantly increased the expression of *CYP24A1* compared to the controls (*p* < 0.001) (Figure 3D).

To test those genes related to DNA base excision repair, we focused on studying the *MBD4, PARP1, WRN*, and *LIG1* genes (Supplementary Figure S3). The expression of those genes did not differ in adipose tissue from lean participants and participants with obesity (*p* < 0.05) (Supplementary Figure S3A). As for the explants, *MBD4* and *LIG1* had higher expression in obese patients compared to lean participants and cancer patients (*p* < 0.05). Furthermore, *PARP1* expression was increased in explants from cancer patients when compared to lean participants and those with obesity (*p* < 0.05) (Supplementary Figure S3B). Finally, adipocytes treated with calcitriol showed increased expression of *MBD4* and PARP1 when compared to the controls (*p* < 0.01 and *p* < 0.05, respectively) (Supplementary Figure S3C).

## 3. Discussion

In the present study, we conducted a complete analysis of the transcriptional and epigenetic profiles of *OGG1* in participants with obesity, as well as in patients with CRC. We found that *OGG1* was upregulated and hypermethylated in adipose tissue from patients with CRC when compared to healthy participants, which was confirmed in adipose tissue explants. In addition, *OGG1* expression was associated with the majority of genes in the BER pathways, suggesting a cooperative mechanism. Furthermore, gene expression in adipose tissue was linked to an increased risk of CRC, while gene expression in whole blood was a promising prognostic biomarker, with higher expression linked to worse survival. These findings revealed dysregulated mechanisms of BER genes in obesity-associated CRC, in which these genes could be potentiated in the obesity state associated with CRC.

Several studies have reported the transcriptional profile of the *OGG1* gene in CRC. For instance, a study reported that *OGG1* was upregulated in adenomas with severe dysplasia and adenocarcinomas compared to normal adjacent tissue [23]. Furthermore, Leguisamo et al. (2017) [24] discovered that *OGG1* expression was higher in 70 tumor tissue samples compared to matched adjacent tissue, which was consistent with our findings. Accordingly, we found that *OGG1* was upregulated in adipose tissue from patients with CRC in comparison with normal participants, indicating increased *OGG1* activity to mitigate the high levels of 8-oxo-2′-deoxyguanosine observed in the cancer context [23]. In contrast, a study conducted by Slyskova et al. (2012) reported decreased transcription levels of *OGG1* in tumor tissue from newly diagnosed patients with sporadic CRC when compared with controls [25]. In addition, another study by Santos et al. (2014) showed that *OGG1* expression was significantly downregulated in tumor samples (stage III and IV) compared to normal tissue and early-stage tumor samples [26]. Therefore, *OGG1* expression could vary depending on confounding variables, as well as other clinical factors. It is important to consider these factors when interpreting the relationship between OGG1 expression levels and CRC.

In terms of *OGG1* promoter methylation, we discovered that the *OGG1* promoter was hypermethylated in adipose tissue from CRC patients compared to healthy participants, which was consistent with the findings of a study conducted by Slyskova et al. (2012) [27]. The latter reported aberrant methylation of *OGG1* in 56% of tumors using microsatellite instability methods, although other methods did not further confirm these findings. It is important to note that the relationship between promoter methylation and gene expression is complex. In some cases, promoter methylation may actually be associated with increased gene expression, particularly if the DNA methylation affects the binding of specific repressor proteins [28]. In this study, we found that the promoter methylation of the *OGG1* gene was increased, along with an increase in gene expression. This finding is interesting because increased promoter methylation is typically associated with decreased gene expression. However, it is possible that the increased methylation in this case may affect the binding of key transcription factors that affect both chromatin accessibility and the initiation of transcription. However, future studies are needed to clarify this observation. However, only a few studies have reported the status of *OGG1* methylation, on which it is relatively premature to make a statement.

As for obesity, *Ogg1* in mice seems to play a protective role against obesity. Accordingly, a study by Sampath et al. (2012) found that *Ogg1^−/−^* mice had increased adiposity and insulin resistance compared to wild-type animals when exposed to a high-fat diet [17]. Specifically, transgenic mice overexpressing human *OGG1* in the mitochondria had significant protection against obesity and adipose tissue inflammation when exposed to a high-fat diet, demonstrating a critical role in energy homeostasis [29]. Furthermore, *Ogg1^−/−^* mice preadipocytes differentiated more efficiently and accumulated more lipids than wild-type preadipocytes. In contrast, *OGG1* overexpression significantly decreased adipogenic differentiation and lipid deposition in both preadipocytes from transgenic mice overexpressing human *OGG1* and 3T3-L1 cells through a significant alteration in cellular PARylation, indicating a role in adipogenesis [30]. In our study, we found that *OGG1* was upregulated in adipose tissue from participants with obesity in comparison with healthy participants and associated with insulin levels, indicating a role in obesity and insulin sensitivity in the human context. This difference might be due to the fact that the adipose tissue of people with obesity has an increased inflammatory profile. Accordingly, *Ogg1*-knockout mice had fewer inflammatory cells and lower expression of cytokines (including IL6 and NF-κB), implying that *OGG1* may play a role in inflammation [31]. Previous murine observations and our results point to a potential role of *OGG1* in protecting and/or resistance against endogenous obesity- and cancer-related oxidative stress, suggesting that this DNA glycosylase may be a promising target for developing DNA glycosylase inhibitors [32]. However, we must remember that age, sex, and stage of CRC are covariables that should be considered when analyzing the *OGG1* expression response [33].

We also found that increased expression of *OGG1* in adipose tissue was associated with an increased risk of having CRC, according to our logistic regression, whereas high expression of *OGG1* in whole blood was associated with an increased risk of worse survival when compared to low *OGG1* expression. Upregulation of *OGG1* in patients with CRC could indicate increased DNA damage. However, even with increased *OGG1* activity, the repair of all of the DNA damage may not be completely achieved, leading to persistent genomic instability, which could increase the risk of cancer progression and metastasis. Most studies found an association between *OGG1* polymorphisms and the risk of CRC. Accordingly, several polymorphisms have been associated with an increased risk of CRC, such as R154H [34], Arg46Gln [35], or Ser326Cys [13,15]. However, controversial results have been found. For example, several studies found that Ser326Cys was associated with a lower risk of CRC [14,36,37], while others found an increased risk [38] or no association, as shown by two meta-analyses published in 2011 (including 12 studies) and 2017 (including 17 studies) [39,40]. These findings were consistent with our results, as we found no link between Ser326Cys polymorphism and an increased risk of CRC. Nevertheless, due to the conflicting results, more studies are needed. However, this mutation was associated with increased circulating oxidative stress markers, suggesting a systemic effect of oxidative stress.

Finally, given that *OGG1* expression in visceral adipose tissue was associated with 25-hydroxyvitamin D, we decided to test this relationship in vitro due to the role of vitamin D in oxidative stress and inflammation [41]. A study by Lan Nan et al. (2014) reported that patients with severe asthma and vitamin D deficiency showed increased *OGG1* protein expression compared to those with vitamin D sufficiency [42]. In contrast, another study by Amirinejab et al. (2021) showed that vitamin D supplements in patients with multiple sclerosis displayed decreased expression of *OGG1* in PBMC [43]. In our study, explants treated with calcitriol (the active metabolite of vitamin D) did not exert any effect on *OGG1* but increased *OGG1* expression in adipocytes. This could be because the activation of CYP24A1 in explants was moderate compared to that in adipocytes, which was hugely increased. Therefore, our experiment with the explants might need more exposure to vitamin D to activate the expression of vitamin-D-targeted genes. Then, these findings can shed light on vitamin D’s role in DNA repair. However, more studies are needed to clarify the mechanisms behind this observation.

## 4. Materials and Methods

### 4.1. Participants and Study Design

Three hundred and ten participants from the “Virgen de la Victoria” University Hospital (Málaga, Spain) were enrolled in this study. Two hundred and thirty were healthy participants, whereas eighty were patients with CRC. Healthy participants were included who underwent hiatus hernia surgery or a cholecystectomy. Patients with CRC underwent surgery with curative intent. The pathological specialist used colonoscopy and biopsy to diagnose patients with CRC. The weight of all participants was stable in the last three months prior to their participation in the study.

The exclusion criteria were patients with diabetes mellitus, acute or chronic inflammatory diseases, renal and infectious diseases, and patients who had received treatment that altered their glucose and lipid metabolism, such as metformin or statins. Furthermore, patients who had changes in other metabolic parameters and who consumed more than 20 g of ethanol per day were excluded. All participants were anonymized prior to typing and gave their written informed consent. The study was performed in accordance with the guidelines of the “Declaration of Helsinki” and approved by the Ethics Committee of “Virgen de la Victoria” University Hospital (Málaga, Spain).

### 4.2. Biochemical Measurement and Sample Obtaining

Blood samples were obtained from all of the participants, and serum samples were extracted by centrifugation at 4000 r.p.m. for 15 min at 4 °C. Fasting glucose, triglycerides, total cholesterol, and high-density lipoprotein (HDL) cholesterol levels were measured using the Dimension Autoanalyzer (Dade Behring Inc., Deerfield, IL, USA). We calculated low-density lipoprotein (LDL) cholesterol using the Friedewald equation [44]. Fasting insulin levels were determined by BioSource International Inc. (Camarillo, CA, USA) using radioimmunoassay methods. The insulin resistance homeostasis model assessment (HOMA-IR) was calculated using the following equation: HOMA-IR = fasting insulin (IU/mL) fasting glucose (mmol/L)/22.5 [45]. Circulating vitamin D levels (25-hydroxyvitamin D (25(OH)D)) were measured using an ELISA kit (Immundiagnostik, Bensheim, Germany).

Epiploic adipose tissue was obtained from healthy participants when they underwent hiatus hernia surgery or cholecystectomy. Epiploic adipose tissue from patients with CRC was obtained during surgery. Adipose tissue samples were frozen in liquid nitrogen until they reached the laboratory. Then, the samples were cleaned in sterile PBS, cut, and stored at −80 °C until analysis.

### 4.3. RNA Extraction and qPCR Analysis

Total RNA was isolated from VAT using an RNeasy Lipid Tissue Mini Kit according to the manufacturer’s instructions (Qiagen GmbH, Hilden, Germany). cDNA was generated from 1000 ng of total RNA using a PrimeScript RT-PCR Kit (Takara Bio USA, Inc., Mountain View, CA, USA) according to the manufacturer’s instructions. The gene expression quantification was carried out using a commercially available TaqMan technology primer/probe mix (Integrated DNA Technologies Inc., Madrid, Spain) for the quantification of 8-oxoguanine DNA glycosylase (*OGG1*, Hs.PT.58.38797078), nuclear factor kappa B subunit 1 (NF-κβ1, Hs.PT.58.20344216), interleukin 1 beta (*IL1β*, Hs.PT.58.1518186), interleukin 6 (IL6, Hs.PT.58.40226675), interleukin 10 (*IL10*, Hs.PT.58.2807216), sirtuin 3 (*SIRT3*, Hs00953477_m1, Thermo Fisher Scientific, Waltham, MA, USA), DNA ligase 1 (*LIG1*, Hs.PT.58.15225811), poly-ADP-ribose polymerase 1 (*PARP1*, Hs.PT.56a.4895683), Werner syndrome helicase (WRN, Hs.PT.58.3388357), methyl-CpG binding domain 4 (MBD4, Hs.PT.58.39947182), carnitine palmitoyltransferase 1A (*CPT1ɑ*, Hs00912671_m1, Thermo Fisher Scientific), cytochrome P450 family 24 subfamily A member 1 (*CYP24A1*, Hs.PT.58.3820691), transcription factor A mitochondrial (*TFAM*, Hs00273372_s1, Thermo Fisher Scientific), and peptidylprolyl isomerase A (*PPIA*, Hs.PT.58v.38887593.g) as an endogenous control. Gene expression was performed by real-time PCR with QuantStudio 6 Pro (Applied Biosystems, Darmstadt, Germany) using Premix Ex Taq™ (Probe qPCR) (Takara Bio USA, Inc., Mountain View, CA, USA), according to the instructions of the manufacturer. Changes in gene expression were calculated using the 2^−ΔCt^ method [44]. The expression results were normalized as the target gene/PPIA ratio.

### 4.4. DNA Extraction and Genotyping

Genomic DNA was extracted from 200 µL of peripheral blood using a QIAamp DNA Blood mini kit (Qiagen GmbH, Hilden, Germany) according to the manufacturer’s instructions, and from VAT using a QIAamp DNA Tissue Kit according to the manufacturer’s instructions (Qiagen GmbH, Hilden, Germany). DNA purity was measured by the A260/A280 and A260/A230 ratios. The information on the *OGG1* rs1052133 SNP was provided by the SNPedia Database System http://www.snpedia.com (accessed on 12 January 2023), dbSNP, and Ensembl [46]. The SNP assays were performed using allele-specific quantitative PCR and QuantStudio 6 Pro (Applied Biosystems, Darmstadt, Germany), using the rhAmp SNP Genotyping System (Integrated DNA Technologies Inc., Madrid, Spain), according to the manufacturer’s instructions. The reaction mixture contained Combined Master Mix and Reporter Mix with Reference Dye, rhSNP Assay Hs.GT.rs.1052133.G.1 (Integrated DNA Technologies Inc., Madrid, Spain), 6 ng of template DNA, and nuclease-free water, making a final volume of 10 µL, according to the manufacturer’s instructions. The allelic discrimination was verified using Design and Analysis Software 2.6.0 (Thermo Fisher Scientific).

### 4.5. DNA Bisulfite Reaction and DNA Methylation Array

To extract the whole methylation of the *OGG1* gene, a genome-wide methylation analysis was conducted. To do this, high-quality genomic DNA samples (500 ng) in the adipose tissue from healthy participants (N = 25) and patients with CRC (N = 29) were treated with bisulfite reagent using the EZ-96 DNA Methylation kit (Zymo Research, Irvine, CA, USA). Subsequently, DNA methylation was analyzed by microarray assays using Infinium Human Methylation 450K bead chip technology (Illumina, San Diego, CA, USA). After obtaining data from the *OGG1* gene, DNA quality was checked, normalized, and filtered using the minfi package [47]. After that, DNA methylation for each CpG site was represented by beta and M values ranging from 0 to 1, corresponding to fully unmethylated and fully methylated, respectively. Specific differentially methylated positions from the *OGG1* gene were extracted from both the promoter region and the whole gene.

### 4.6. Cell Culture and Adipose Tissue Explants

Human mesenchymal stem cells from adipose tissue (hMSC-AT, PromoCell C-12977) were seeded at a density of 15 × 10^3^ cells/well in 6-well plates for three days under standard culture conditions. After this period, the expansion medium was replaced by means of induction (72 h) and adipogenic differentiation to adipocytes (18 days) for 21 days. After that, we treated adipocytes for each condition with vehicle (DMSO) and 0.5 µM of calcitriol for 24 h. The experiments were conducted in three independent replicates using N = 1.

As for adipose tissue explants, epiploic adipose tissue from 3 healthy lean participants, 3 healthy participants with obesity, and 3 patients with CRC were used in this study. The adipose tissue was cut into 5–10 mg pieces to do this. Then, these pieces were incubated in PBS supplemented with 5% BSA for 30 min. After that, a serum-free medium supplemented with 1% P/S and 200 mmol/L of L-glutamine was added to the pieces and kept at 37 °C. For the vehicle, DMSO was added to the medium. For treatment, 1 µM calcitriol was added to the medium. After 24 h of treatment, RNA was extracted using the RNeasy Lipid Tissue Mini Kit (Qiagen GmbH, Hilden, Germany) according to the manufacturer’s instructions [48]. cDNA was obtained as previously described.

### 4.7. Statistical and Bioinformatic Analysis

Continuous variables are presented as means ± standard deviations. The Welch (parametric) or Wilcoxon (non-parametric) two-sample tests were used to determine differences between healthy participants and patients with CRC. Differences in qualitative variables and the Hardy–Weinberg equilibrium (HWE) were tested using the χ^2^ test. The Kruskal–Wallis test was used to evaluate the differences between groups for non-parametric variables. Pearson’s correlation coefficients were calculated for parametric variables, or Spearman’s correlation for non-parametric variables. Univariate and multivariate logistic regression models estimated hazard ratios to assess the association of the *OGG1* methylation and expression with increased CRC risk. Analyses and graphical representations were created using R 4.2.1 software (Integrated Development for R. RStudio, PBC, Boston, MA, USA) and GraphPad Prism, and the significance threshold was set at *p* < 0.05.

## 5. Conclusions

*OGG1* expression and methylation were upregulated in adipose patients with CRC compared to healthy participants. *OGG1* seems to be a risk factor for CRC, since increased *OGG1* expression in VAT was associated with an increased risk of CRC, while high *OGG1* expression in whole blood was associated with poor overall survival. Furthermore, calcitriol treatment increased *OGG1* levels in adipocytes, suggesting a protective link between vitamin D and DNA repair in adipose tissue. Therefore, *OGG1* is a promising biomarker in obesity-related CRC and can act as a protective factor against CRC. However, further studies are needed to understand this relationship better.

## Figures and Tables

**Table 1:** The baseline characteristics of the participants included in the study.

Variable	All	Healthy Participants	Patients with CRC	*p*
	N = 310	N = 230	N = 80	
Age (years)	54.5 (15.0)	50.1 (13.8)	67.2 (10.3)	<0.001 *
Sex (males/females)	159/151	103/127	56/24	<0.001 *
BMI (Kg/m^2^)	28.9 (7.33)	29.5 (8.09)	27.2 (4.06)	0.001 *
Glucose (mg/dL)	105 (35.2)	98.3 (20.3)	125 (55.9)	<0.001 *
HOMAIR	2.38 (1.82)	2.50 (1.76)	2.01 (1.95)	0.054
Total cholesterol (mg/dL)	196 (44.7)	205 (42.7)	173 (41.3)	<0.001 *
Triglycerides (mg/dL)	134 (67.4)	123 (58.5)	163 (79.6)	<0.001 *
LDL (mg/dL)	120 (34.7)	127 (32.9)	104 (33.8)	<0.001 *
HDL (mg/dL)	50.0 (15.3)	53.4 (14.4)	41.2 (14.1)	<0.001 *
25-Hydroxyvitamin D (ng/mL)	39.1 (18.9)	43.5 (20.2)	30.8 (12.8)	<0.001 *
*OGG1* Ser302Cys				0.174
C/C	146 (65.8%)	100 (66.2%)	46 (64.8%)	
C/G	61 (27.5%)	44 (29.1%)	17 (23.9%)	
G/G	15 (6.76%)	7 (4.64%)	8 (11.3%)	

Data are expressed as means ± standard deviations or percentages. Asterisks indicate significant differences between groups, according to Welch’s two-sample tests (* *p* < 0.05). The chi-squared test was used for variables expressed as percentages (* *p* < 0.05). Abbreviations: 25(OH)D: 25-hydroxyvitamin D; BMI: body mass index; CRC: colorectal cancer; HOMA-IR: homeostasis model of insulin resistance; HDL: high-density lipoprotein; LDL: low-density lipoprotein; OGG1: 8-oxoguanine DNA glycosylase 1.

**Table 2:** Multiple regression analysis of the *OGG1* gene as a predictor of the risk of colorectal cancer incidence.

Variables	β (SE) R^2^ = 0.41, *p* < 0.001	β (SE) R^2^ = 0.30, *p* < 0.001	β (SE) R^2^ = 0.44, *p* < 0.001	β (SE) R^2^ = 0.40, *p* < 0.001
Age (years)	0.16 (0.00) ***	0.01 (0.00) ***	0.02 (0.00) ***	0.02 (0.00) ***
Sex (males/females)	−0.23 (0.11)	−0.17 (0.08) *	−0.13 (0.12)	−0.15 (0.12)
BMI (kg/m^2^)	0.00 (0.01)	−0.02 (0.01) *	−0.01 (0.011)	−0.01 (0.01)
*OGG1* expression in VAT	NA	7.21 (3.09) *	NA	NA
*OGG1* expression in whole blood	0.91 (0.55)	NA	NA	NA
Promoter *OGG1* methylation	NA	NA	10.22 (5.53)	NA
Body *OGG1* methylation	NA	NA	NA	−3.16 (6.91)

Data are expressed as β (standard error). Data adjusted for sex, age, and BMI. Asterisks indicate significant values according to the test (* *p* < 0.05; *** *p* < 0.001). Abbreviations: OGG1: 8-oxoguanine DNA glycosylase 1; BMI: body mass index.

## Data Availability

All data generated or analyzed during this study are included in this published article.

## Supplementary Materials

The following supporting information can be downloaded at: https://www.mdpi.com/article/10.3390/ijms24065488/s1.

## References

[1] Cabrera-Mulero A., Crujeiras A.B., Izquierdo A.G., Torres E., Ayers D., Casanueva F.F., Tinahones F.J., Morcillo S., Macias-Gonzalez M. (2019). Novel *SFRP2* DNA Methylation Profile Following Neoadjuvant Therapy in Colorectal Cancer Patients with Different Grades of BMI. J. Clin. Med..

[2] Sung H., Ferlay J., Siegel R.L., Laversanne M., Soerjomataram I., Jemal A., Bray F. (2021). Global Cancer Statistics 2020: GLOBOCAN Estimates of Incidence and Mortality Worldwide for 36 Cancers in 185 Countries. CA Cancer J. Clin..

[3] Gathirua-Mwangi W.G., Monahan P., Song Y., Zollinger T.W., Champion V.L., Stump T.E., Imperiale T.F. (2017). Changes in Adult BMI and Waist Circumference Are Associated with Increased Risk of Advanced Colorectal Neoplasia. Dig. Dis. Sci..

[4] Silveira E.A., Vaseghi G., Santos A.S.D.C., Kliemann N., Masoudkabir F., Noll M., Mohammadifard N., Sarrafzadegan N., De Oliveira C. (2020). Visceral Obesity and Its Shared Role in Cancer and Cardiovascular Disease: A Scoping Review of the Pathophysiology and Pharmacological Treatments. Int. J. Mol. Sci..

[5] Srinivas U.S., Tan B.W.Q., Vellayappan B.A., Jeyasekharan A.D. (2019). ROS and the DNA damage response in cancer. Redox Biol..

[6] Tian G., Katchur S.R., Jiang Y., Briand J., Schaber M., Kreatsoulas C., Schwartz B., Thrall S., Davis A.M., Duvall S. (2022). Small molecule-mediated allosteric activation of the base excision repair enzyme 8-oxoguanine DNA glycosylase and its impact on mitochondrial function. Sci. Rep..

[7] Kabziński J., Majsterek I. (2022). Association of base excision repair pathway genes OGG1, XRCC1 and mutyh polymorphisms and the level of 8-oxo-guanine with increased risk of colorectal cancer occurrence. Int. J. Occup. Med. Environ. Health.

[8] Obtułowicz T., Swoboda M., Speina E., Gackowski D., Rozalski R., Siomek A., Janik J., Janowska B., Cieśla J.M., Jawien A. (2010). Oxidative stress and 8-oxoguanine repair are enhanced in colon adenoma and carcinoma patients. Mutagenesis.

[9] D’Augustin O., Huet S., Campalans A., Radicella J. (2020). Lost in the Crowd: How Does Human 8-Oxoguanine DNA Glycosylase 1 (OGG1) Find 8-Oxoguanine in the Genome?. Int. J. Mol. Sci..

[10] Ba X., Boldogh I. (2017). 8-Oxoguanine DNA glycosylase 1: Beyond repair of the oxidatively modified base lesions. Redox Biol..

[11] Karahalil B., Engin A.B., Coskun E. (2012). Could 8-oxoguanine DNA glycosylase 1 Ser326Cys polymorphism be a biomarker of susceptibility in cancer?. Toxicol. Ind. Health.

[12] Sliwinski T., Krupa R., Wisniewska-Jarosinska M., Pawlowska E., Lech J., Chojnacki J., Blasiak J. (2009). Common Polymorphisms in the XPD and hOGG1 Genes Are Not Associated with the Risk of Colorectal Cancer in a Polish Population. Tohoku J. Exp. Med..

[13] Kabzinski J., Walczak A., Dziki A., Mik M., Majsterek I. (2018). Impact of the Ser326Cys polymorphism of the OGG1 gene on the level of oxidative DNA damage in patients with colorectal cancer. Ann. Surg..

[14] Su Y., Xu A., Zhu J. (2013). The effect of oxoguanine glycosylase 1 rs1052133 polymorphism on colorectal cancer risk in Caucasian population. Tumor Biol..

[15] Kang S.W., Kim S.K., Park H.J., Chung J.-H., Ban J.Y. (2017). Human 8-oxoguanine DNA glycosylase gene polymorphism (Ser326Cys) and cancer risk: Updated meta-analysis. Oncotarget.

[16] Leu M., Riebeling T., Dröge L., Hubert L., Guhlich M., Wolff H., Brockmöller J., Gaedcke J., Rieken S., Schirmer M. (2021). 8-Oxoguanine DNA Glycosylase (OGG1) Cys326 Variant: Increased Risk for Worse Outcome of Patients with Locally Advanced Rectal Cancer after Multimodal Therapy. Cancers.

[17] Sampath H., Vartanian V., Rollins M.R., Sakumi K., Nakabeppu Y., Lloyd R.S. (2012). 8-Oxoguanine DNA Glycosylase (OGG1) Deficiency Increases Susceptibility to Obesity and Metabolic Dysfunction. PLoS ONE.

[18] Ouni M., Schürmann A. (2020). Epigenetic contribution to obesity. Mamm. Genome.

[19] Włodarczyk M., Nowicka G. (2019). Obesity, DNA Damage, and Development of Obesity-Related Diseases. Int. J. Mol. Sci..

[20] Usman M., Volpi E.V. (2018). DNA damage in obesity: Initiator, promoter and predictor of cancer. Mutat. Res. Mol. Mech. Mutagen..

[21] Kompella P., Vasquez K.M. (2019). Obesity and cancer: A mechanistic overview of metabolic changes in obesity that impact genetic instability. Mol. Carcinog..

[22] Pike J.W., Meyer M.B. (2012). Regulation of mouse Cyp24a1 expression via promoter-proximal and downstream-distal enhancers highlights new concepts of 1,25-dihydroxyvitamin D3 action. Arch. Biochem. Biophys..

[23] Sæbø M., Skjelbred C.F., Nexø B.A., Wallin H., Hansteen I.-L., Vogel U., Kure E.H. (2006). Increased mRNA expression levels of ERCC1, OGG1 and RAI in colorectal adenomas and carcinomas. BMC Cancer.

[24] Leguisamo N.M., Gloria H.C., Kalil A.N., Martins T.V., Azambuja D.B., Meira L.B., Saffi J. (2017). Base excision repair imbalance in colorectal cancer has prognostic value and modulates response to chemotherapy. Oncotarget.

[25] Slyskova J., Naccarati A., Pardini B., Polakova V., Vodickova L., Smerhovsky Z., Levy M., Lipska L., Liska V., Vodicka P. (2012). Differences in nucleotide excision repair capacity between newly diagnosed colorectal cancer patients and healthy controls. Mutagenesis.

[26] Santos J.C., Funck A., Silva-Fernandes I.J.L., Rabenhorst S.H.B., Martinez C.A.R., Ribeiro M.L. (2014). Effect of APE1 T2197G (Asp148Glu) Polymorphism on APE1, XRCC1, PARP1 and OGG1 Expression in Patients with Colorectal Cancer. Int. J. Mol. Sci..

[27] Slyskova J., Korenkova V., Collins A.R., Prochazka P., Vodickova L., Svec J., Lipska L., Levy M., Schneiderova M., Liska V. (2012). Functional, Genetic, and Epigenetic Aspects of Base and Nucleotide Excision Repair in Colorectal Carcinomas. Clin. Cancer Res..

[28] Dhar G.A., Saha S., Mitra P., Chaudhuri R.N. (2021). DNA methylation and regulation of gene expression: Guardian of our health. Nucl..

[29] Komakula S.S.B., Tumova J., Kumaraswamy D., Burchat N., Vartanian V., Ye H., Dobrzyn A., Lloyd R.S., Sampath H. (2018). The DNA Repair Protein OGG1 Protects Against Obesity by Altering Mitochondrial Energetics in White Adipose Tissue. Sci. Rep..

[30] Komakula S., Blaze B., Ye H., Dobrzyn A., Sampath H. (2021). A Novel Role for the DNA Repair Enzyme 8-Oxoguanine DNA Glycosylase in Adipogenesis. Int. J. Mol. Sci..

[31] Li G., Yuan K., Yan C., Fox J., Gaid M., Breitwieser W., Bansal A.K., Zeng H., Gao H., Wu M. (2012). 8-Oxoguanine-DNA glycosylase 1 deficiency modifies allergic airway inflammation by regulating STAT6 and IL-4 in cells and in mice. Free. Radic. Biol. Med..

[32] Visnes T., Grube M., Hanna B.M.F., Benitez-Buelga C., Cázares-Körner A., Helleday T. (2018). Targeting BER enzymes in cancer therapy. DNA Repair.

[33] Wele P., Wu X., Shi H. (2022). Sex-Dependent Differences in Colorectal Cancer: With a Focus on Obesity. Cells.

[34] Kim I.-J., Ku J.-L., Kang H.C., Park J.-H., Yoon K.-A., Shin Y., Park H.-W., Jang S.G., Lim S.-K., Han S.Y. (2004). Mutational analysis of OGG1, MYH, MTH1 in FAP, HNPCC and sporadic colorectal cancer patients: R154H OGG1 polymorphism is associated with sporadic colorectal cancer patients. Hum. Genet..

[35] Garre P., Briceño V., Xicola R.M., Doyle B.J., de la Hoya M., Sanz J., Llovet P., Pescador P., Puente J., Díaz-Rubio E. (2011). Analysis of the Oxidative Damage Repair Genes *NUDT1*, *OGG1*, and *MUTYH* in Patients from Mismatch Repair Proficient HNPCC Families (MSS-HNPCC). Clin. Cancer Res..

[36] Hansen R., Sæbø M., Skjelbred C.F., Nexø B.A., Hagen P.C., Bock G., Lothe I.M.B., Johnson E., Aase S., Hansteen I.-L. (2005). GPX Pro198Leu and OGG1 Ser326Cys polymorphisms and risk of development of colorectal adenomas and colorectal cancer. Cancer Lett..

[37] Przybylowska K., Kabzinski J., Sygut A., Dziki L., Dziki A., Majsterek I. (2013). An association selected polymorphisms of XRCC1, OGG1 and MUTYH gene and the level of efficiency oxidative DNA damage repair with a risk of colorectal cancer. Mutat. Res. Mol. Mech. Mutagen..

[38] Moreno V., Gemignani F., Landi S., Gioia-Patricola L., Chabrier A., Blanco I., González S., Guino E., Capellà G., Canzian F. (2006). Polymorphisms in Genes of Nucleotide and Base Excision Repair: Risk and Prognosis of Colorectal Cancer. Clin. Cancer Res..

[39] Zhang Y., He B.-S., Pan Y.-Q., Xu Y.-Q., Wang S.-K. (2011). Association of OGG1 Ser326Cys polymorphism with colorectal cancer risk: A meta-analysis. Int. J. Color. Dis..

[40] Aggarwal N., Donald N.D., Malik S., Selvendran S.S., McPhail M., Monahan K.J. (2017). The Association of Low-Penetrance Variants in DNA Repair Genes with Colorectal Cancer: A Systematic Review and Meta-Analysis. Clin. Transl. Gastroenterol..

[41] Gaiani F., Marchesi F., Negri F., Greco L., Malesci A., De’Angelis G., Laghi L. (2021). Heterogeneity of Colorectal Cancer Progression: Molecular Gas and Brakes. Int. J. Mol. Sci..

[42] Lan N., Luo G., Yang X., Cheng Y., Zhang Y., Wang X., Wang X., Xie T., Li G., Liu Z. (2014). 25-Hydroxyvitamin D3-Deficiency Enhances Oxidative Stress and Corticosteroid Resistance in Severe Asthma Exacerbation. PLoS ONE.

[43] Amirinejad R., Shirvani-Farsani Z., Gargari B.N., Sahraian M.A., Soltani B.M., Behmanesh M. (2021). Vitamin D changes expression of DNA repair genes in the patients with multiple sclerosis. Gene.

[44] Friedewald W.T., Levy R.I., Fredrickson D.S. (1972). Estimation of the Concentration of Low-Density Lipoprotein Cholesterol in Plasma, Without Use of the Preparative Ultracentrifuge. Clin. Chem..

[45] Levy J.C., Matthews D.R., Hermans M.P. (1998). Correct Homeostasis Model Assessment (HOMA) Evaluation Uses the Computer Program. Diabetes Care.

[46] Zerbino D.R., Achuthan P., Akanni W., Amode M.R., Barrell D., Bhai J., Billis K., Cummins C., Gall A., Girón C.G. (2018). Ensembl. Nucleic Acids Res..

[47] Aryee M.J., Jaffe A.E., Corrada-Bravo H., Ladd-Acosta C., Feinberg A.P., Hansen K.D., Irizarry R.A. (2014). Minfi: A flexible and comprehensive Bioconductor package for the analysis of Infinium DNA methylation microarrays. Bioinformatics.

[48] Clemente-Postigo M., Muñoz-Garach A., Serrano M., Garrido-Sánchez L., Bernal-López M.R., Fernández-García D., Moreno-Santos I., Garriga N., Castellano-Castillo D., Camargo A. (2015). Serum 25-Hydroxyvitamin D and Adipose Tissue Vitamin D Receptor Gene Expression: Relationship With Obesity and Type 2 Diabetes. J. Clin. Endocrinol. Metab..

