# Supplementary material for: 8-Oxoguanine DNA Glycosylase 1 Upregulation as a Risk Factor for Obesity and Colorectal Cancer
Source: Int J Mol Sci. 2023 Mar 13;24(6):5488. doi: 10.3390/ijms24065488 (PMC10052644; doi:10.3390/ijms24065488)
Supplement: Supplementary file 1 [file ijms-24-05488-s001.zip › Supplementary figure captions.pdf]

Supplementary Materials figure captions:

Supplementary Figure S1. A) Validation of OGG1 expression in PBMCs comparing healthy participants (N=24) and patients with CRC (N=33). **B)** Normalized gene expression of *OGG1* comparing healthy lean participants (N=13) with participants with overweight/obese (N=41). In addition, we compare lean (N=22) and overweight/obese patients with CRC (N=43). **C)** Promoter methylation of OGG1 comparing healthy participants (N=24) and patients with CRC (N=23). The promoter contained the following CpG sites: cg11841349, cg14201528, cg15357639, cg17285536, cg17319894, cg19391888, cg25415932, cg05439191. **D)** Pearson correlation analysis between Body *OGG1* methylation and *OGG1* expression ($p<0.05$). The body region contained the following CpG sites: cg01396022, cg06730340, cg09511906, cg11888747, cg12353927, cg13896608, cg14558573, cg15771477, cg23243617, cg25949513, cg12684175, cg16943783, cg20782816. Gene expression was normalized using the PPIA gene and the formula: 2-ΔCt. **E)** Receiver operating characteristic (ROC) for the logistic model. Data were adjusted for age, sex, and BMI. **F)** Kaplan–Meier estimate for overall survival according to gene expression of *OGG1* (low vs. high under the median value) in adipose tissue. *Abbreviations:* AUC: Area under the curve; CRC: Colorectal cancer; OGG1: OGG1 8-Oxoguanine DNA Glycosylase 1; Ow/Ob: Overweight/Obese; The asterisks indicate significant values according to the test (*p<0.05; **p<0.01 ***p<0.001).

Supplementary Figure S2. Participants were divided between genotypes, being CC vs. CG/GG, and compared A) HbA1c, **B)** Transferrin, **C)** Ferritin, and **D)** Alkaline phosphatase (ALP). The Welch two (Parametric) or Wilcoxon (Non-parametric) sample tests were used to determine differences between variables. The asterisks indicate significant values according to the test (*p<0.05; **p<0.01 ***p<0.001). Explants were treated with 1μM of calcitriol for 24 hours. Total RNA was extracted, and gene expression was measured. Normalized gene expression of **E)** OGG1 and **F)** CYP24A1 was measured by comparing explants from healthy lean participants (N=3), healthy obese participants (N=3), and patients with CRC (N =4). The asterisks indicate significant values according to the test (*p<0.05; **p<0.01 ***p<0.001). Gene expression was normalized using the PPIA gene and the formula: 2-ΔCt.

Supplementary Figure S3. A) Participants were divided under BMI, being healthy lean vs. overweight/obese participants. Gene expression of MBD4, PARP1, WRN, and LIG1 were measured. **B)** Explants were treated with 1μM of calcitriol for 24 hours. Total RNA was extracted, and gene expression was measured. Gene expression of MBD4, PARP1, WRN, and LIG1 were measured by comparing explants from healthy lean participants (N=3), healthy obese participants (N=3), and patients with CRC (N =4). **C)**Adipocytes were treated with 0.5 μM of calcitriol for 24 hours. Total RNA was extracted, and Gene expression of MBD4, PARP1, WRN, and LIG1 was measured. The asterisks indicate significant values according to the test (*p<0.05; **p<0.01 ***p<0.001). Gene expression was normalized using the PPIA gene and the formula: 2-ΔCt. The asterisks indicate significant values according to the test (*p<0.05; **p<0.01 ***p<0.001). Gene expression was normalized using the PPIA gene and the formula: 2-ΔCt.
