# Supplementary material for: 8-Oxoguanine DNA Glycosylase 1 Upregulation as a Risk Factor for Obesity and Colorectal Cancer
Source: Int J Mol Sci. 2023 Mar 13;24(6):5488. doi: 10.3390/ijms24065488 (PMC10052644; doi:10.3390/ijms24065488)
Supplement: Supplementary file 1 [file ijms-24-05488-s001.zip › Supplementary Table S1.pdf]

Supplementary Table S1. Baseline table of the participants included in the study under obesity state.

	Healthy participants			Patients with CRC		
	**Lean** *N=84*	**Overweight / Obese** *N=146*	***p***	**Lean** *N=26*	**Overweight / Obese** *N=54*	***p***
Age (years)	47.3 (12.9)	51.6 (14.0)	0.075	66.0 (12.8)	67.8 (8.94)	0.939
Sex (Males/Females)	35/49	68/78	0.672	18/8	38/16	1.000
BMI (Kg/m^2)	22.8 (1.80)	33.4 (7.77)	<0.001*	23.2 (1.64)	29.1 (3.39)	<0.001*
Glucose (mg/dL)	92.9 (17.5)	101 (21.2)	0.252	133 (81.2)	121 (39.6)	0.414
HOMAIR	1.74 (1.25)	2.93 (1.86)	<0.001*	1.38 (1.30)	2.30 (2.15)	0.144
Total cholesterol (mg/dL)	201 (37.7)	207 (45.1)	0.764	179 (46.7)	170 (38.8)	0.849
Triglycerides (mg/dL)	106 (63.1)	132 (54.2)	0.035	145 (75.2)	171 (80.9)	0.329
LDL (mg/dL)	122 (31.2)	129 (33.6)	0.412	109 (36.4)	101 (32.7)	0.782
HDL (mg/dL)	57.4 (14.7)	51.4 (13.9)	0.023*	43.2 (14.0)	40.2 (14.2)	0.823
25-hydroxyvitamin D (ng/mL)	48.5 (22.7)	41.2 (18.6)	0.145	31.3 (15.0)	30.6 (11.9)	0.999
OGG1 Ser302Cys			0.735			0.561
C/C	39 (63.9%)	61 (67.8%)		17 (73.9%)	29 (61.7%)	
C/G	18 (29.5%)	26 (28.9%)		3 (13.0%)	13 (27.7%)	
G/G	4 (6.56%)	3 (3.33%)		3 (13.0%)	5 (10.6%)	

Data are expressed as mean ± standard deviations or percentages. Asterisk indicates a significant difference between groups, according to Welch´s two-sample tests (*p<0.05). Chi-squared test was used for variables expressed as percentages (*p<0.05). *Abbreviations*: 25(OH)D: 25-hydroxyvitamin D; BMI: Body mass index; CRC: colorectal cancer; HDL: High-density lipoprotein; HOMA-IR: homeostasis model of insulin resistance; LDL: Low-density lipoprotein; OGG1: 8-OxoGuanine DNA Glycosylase 1.
