# Supplementary figures and images for: 8-Oxoguanine DNA Glycosylase 1 Upregulation as a Risk Factor for Obesity and Colorectal Cancer
Source: Int J Mol Sci. 2023 Mar 13;24(6):5488. doi: 10.3390/ijms24065488 (PMC10052644; doi:10.3390/ijms24065488)

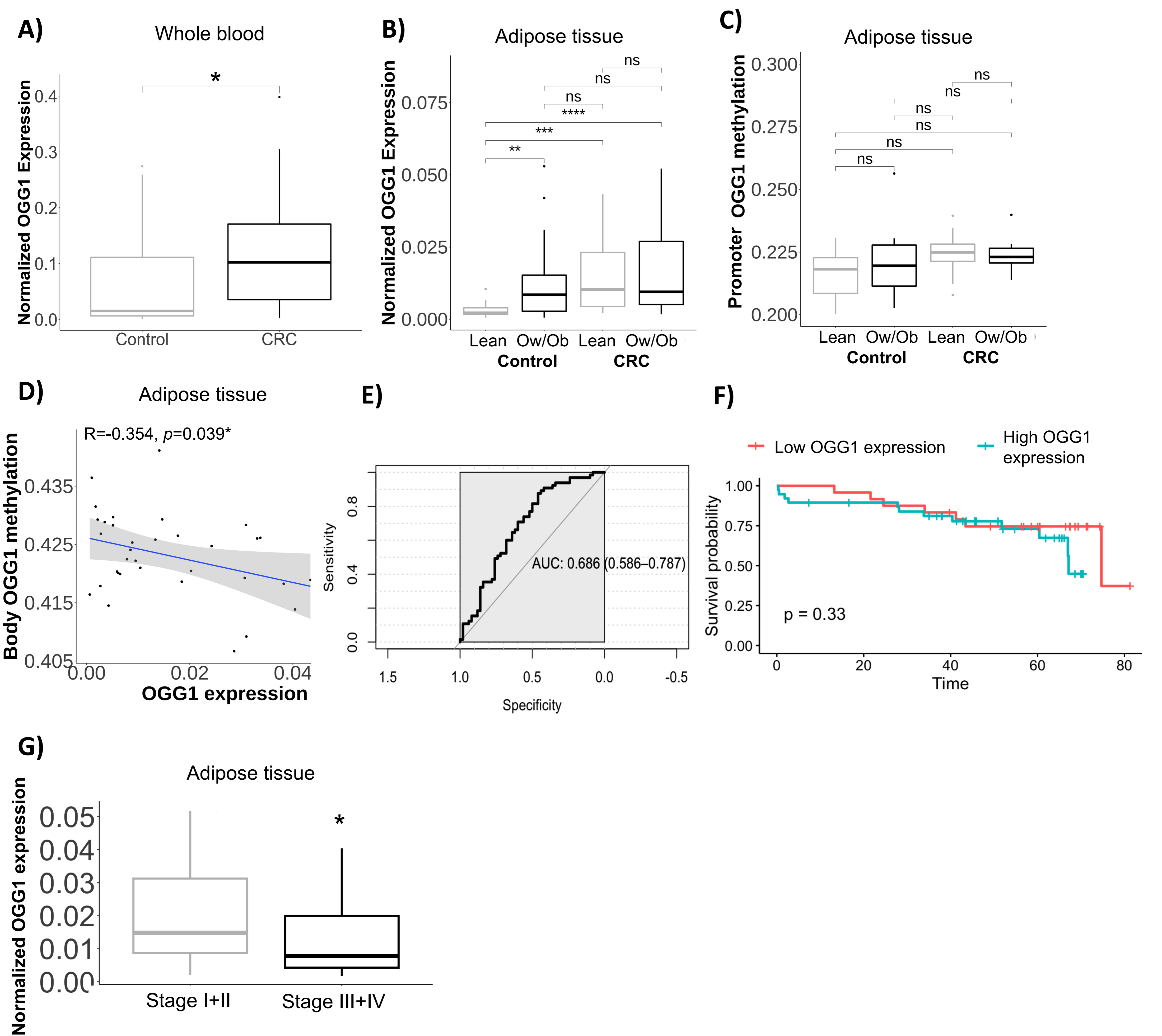

Supplement: Supplementary file 1 [file ijms-24-05488-s001.zip › Supplementary Figure S1.pdf]

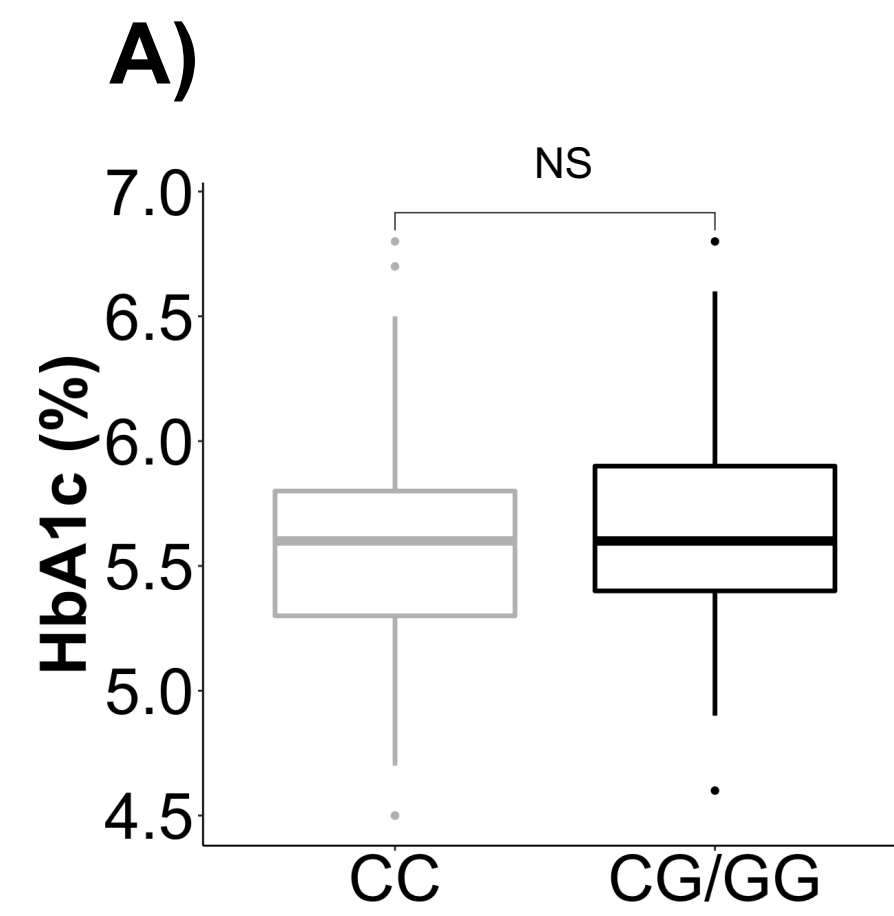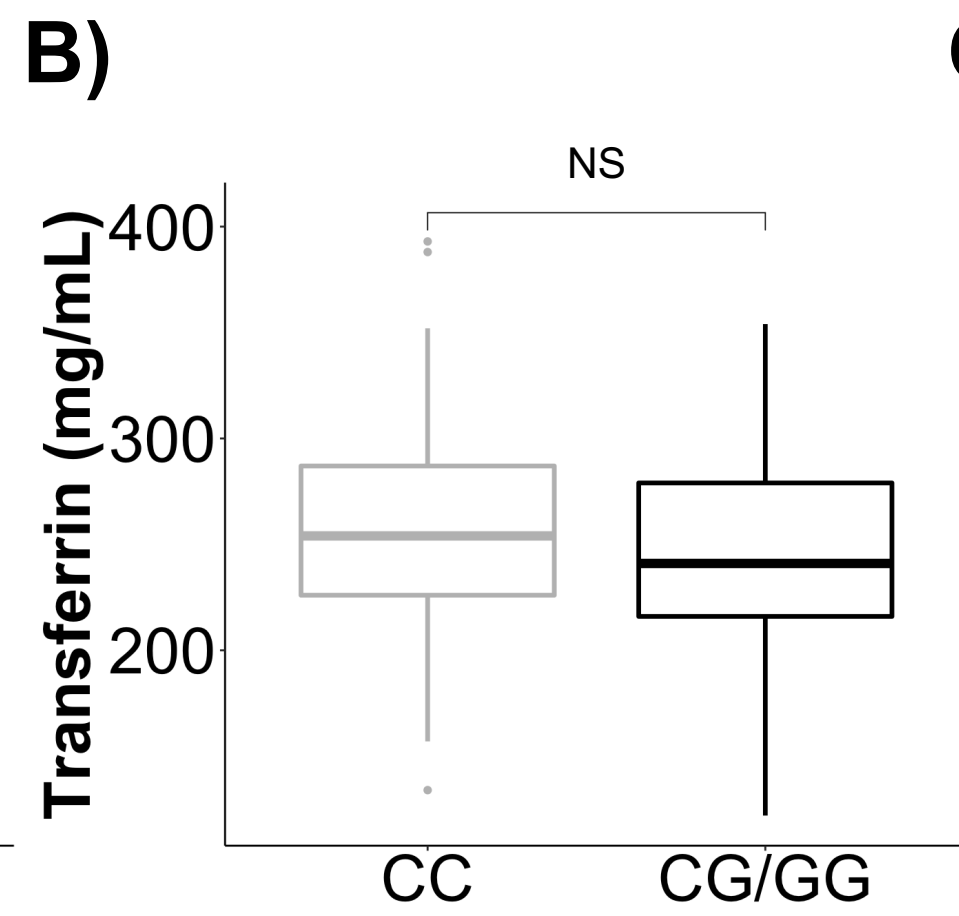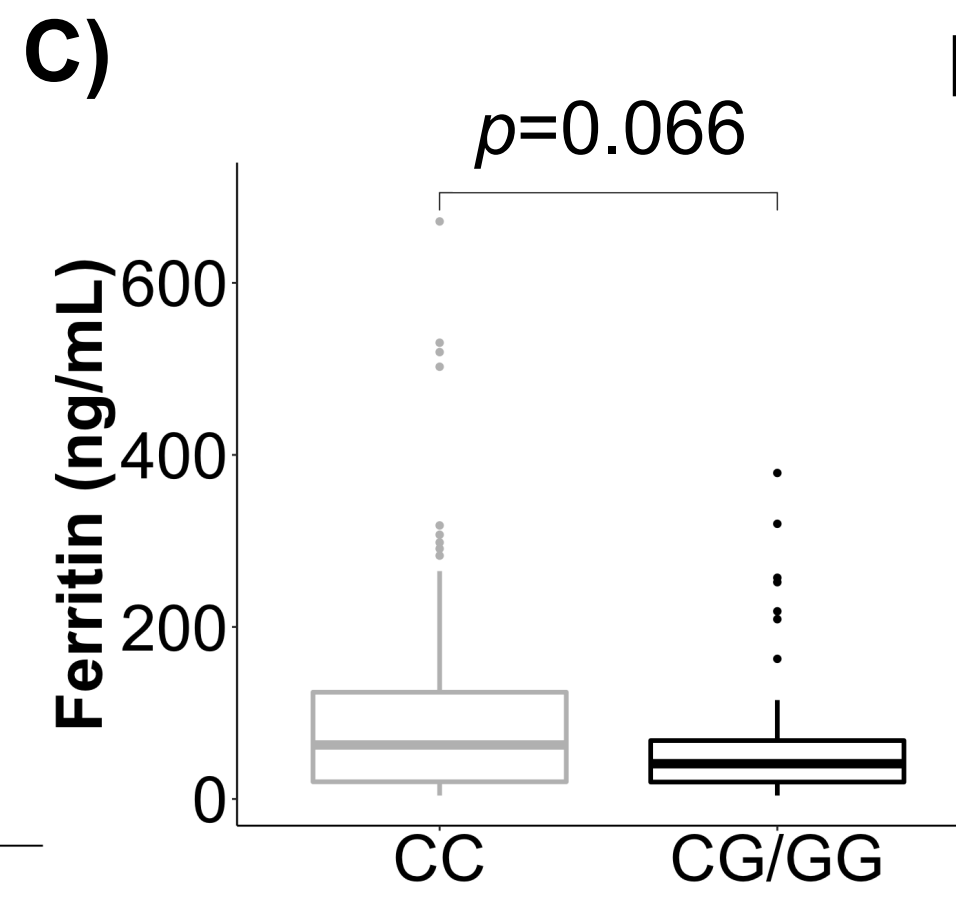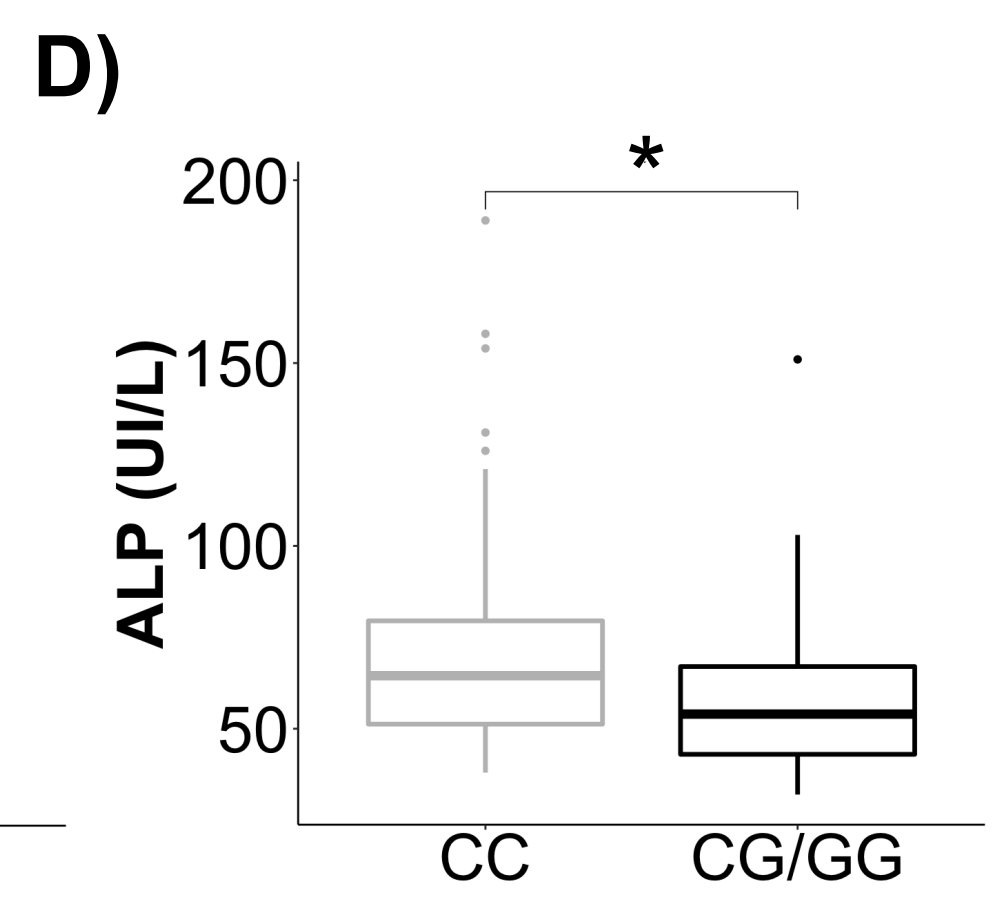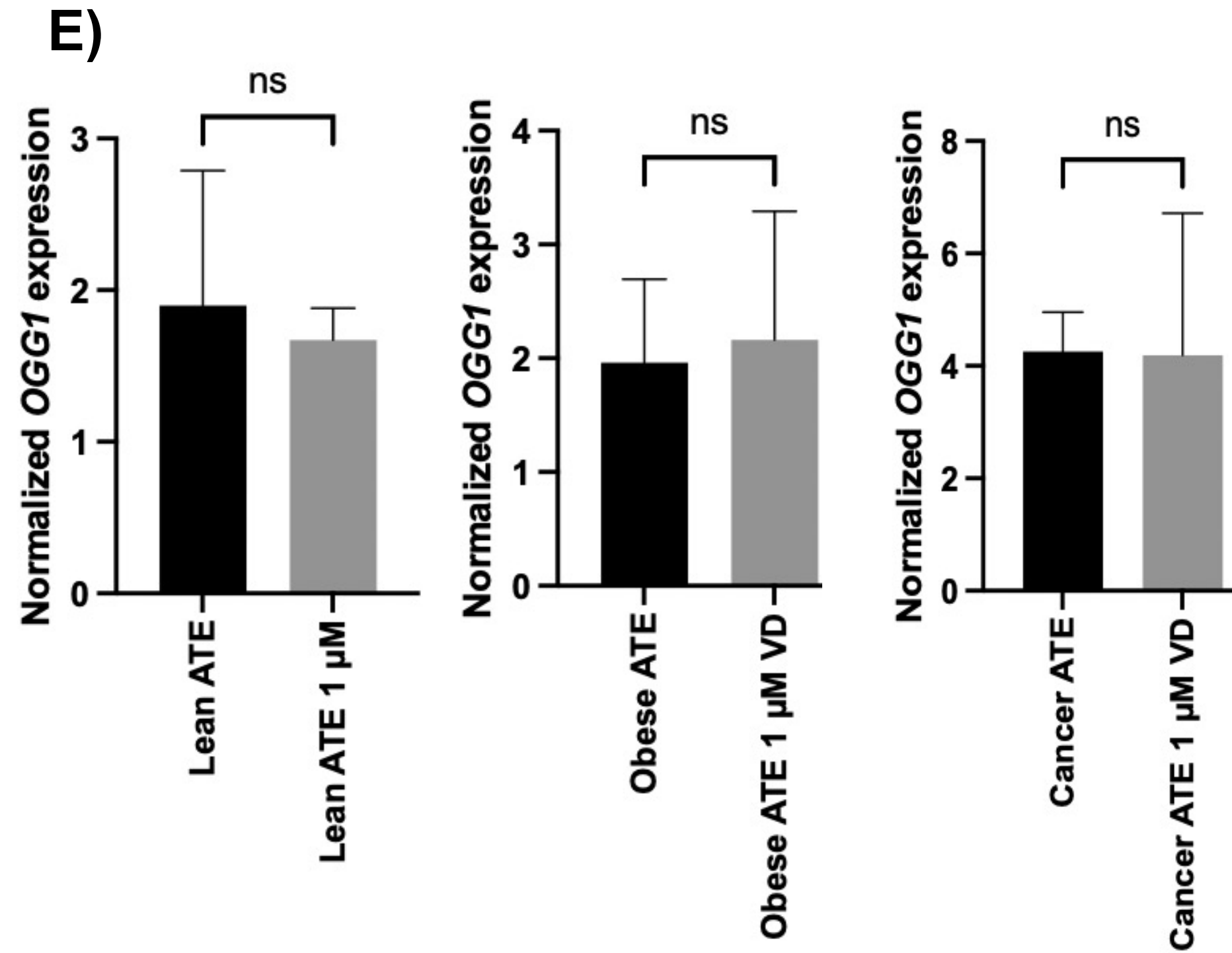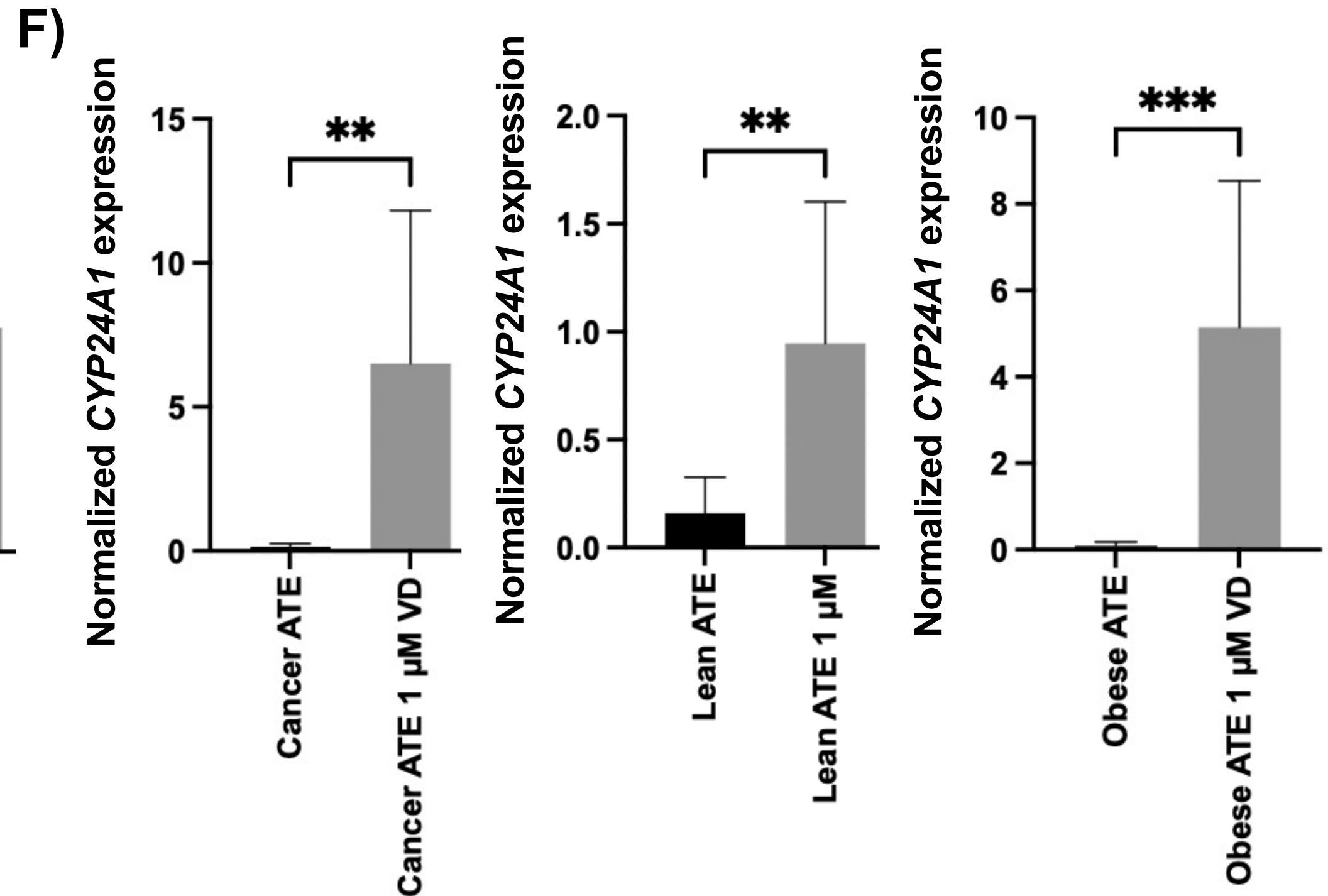

Supplement: Supplementary file 1 [file ijms-24-05488-s001.zip › Supplementary Figure S2.pdf]

**A)**

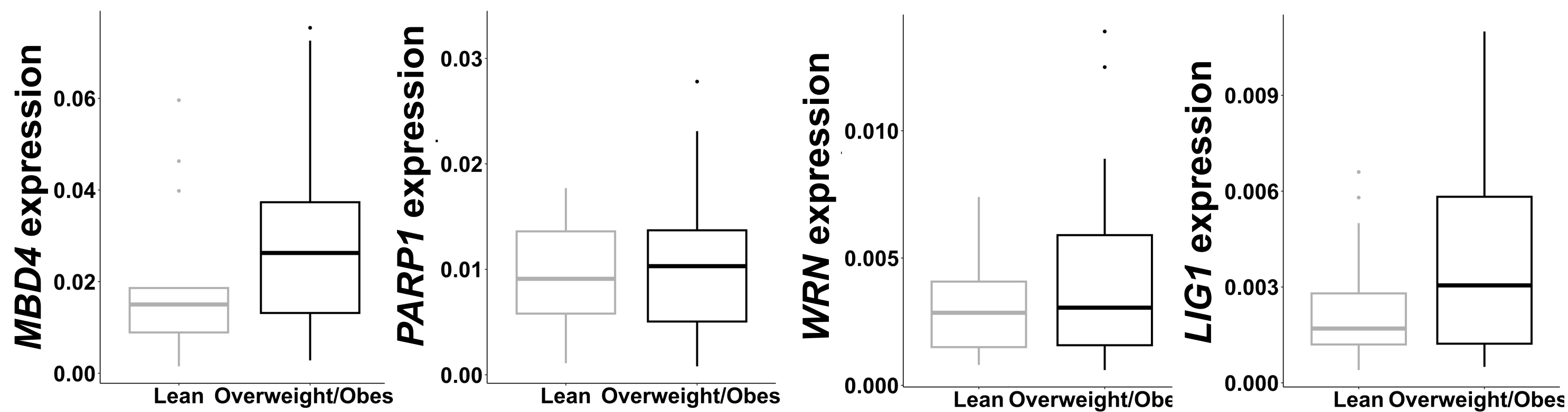

**B)**

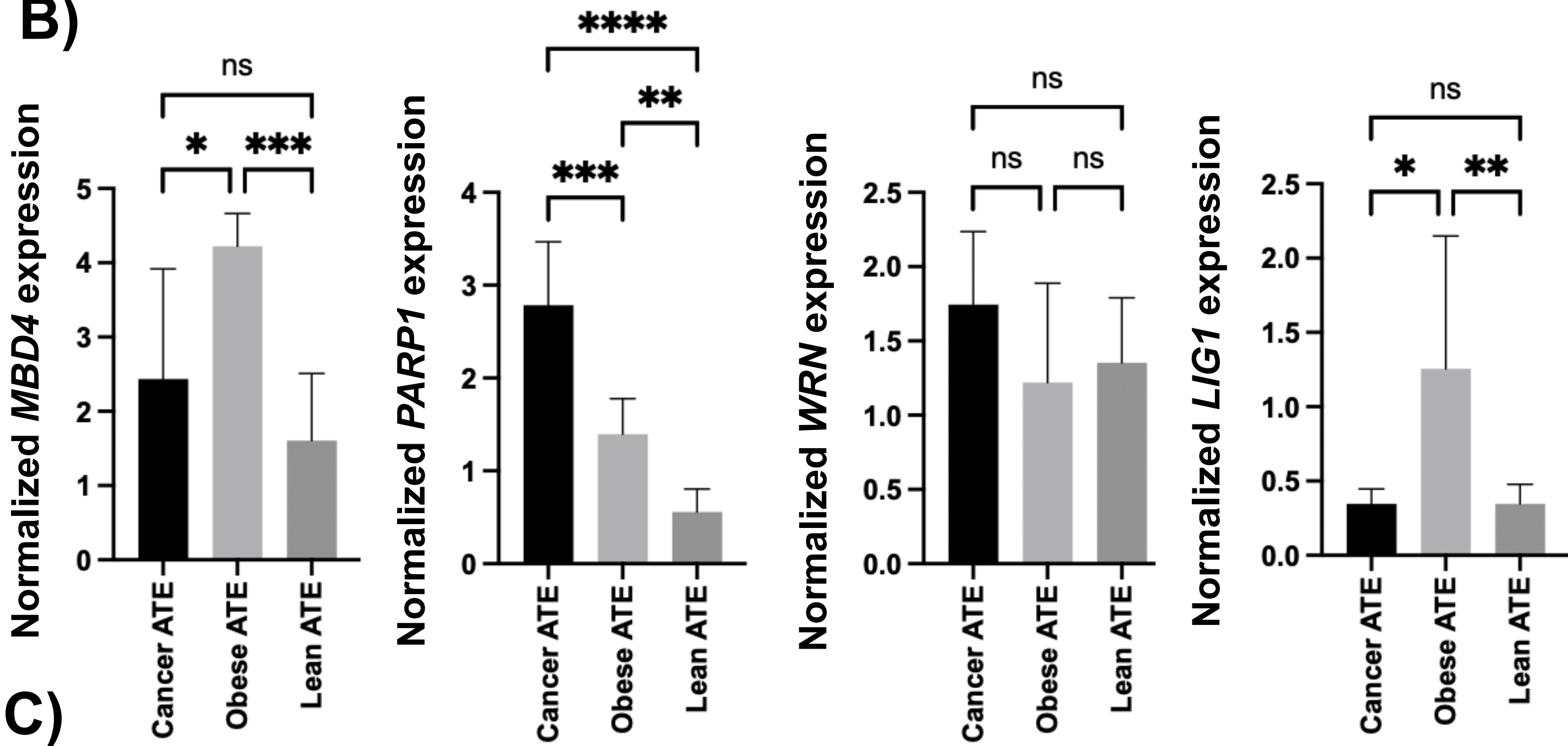

**C)**

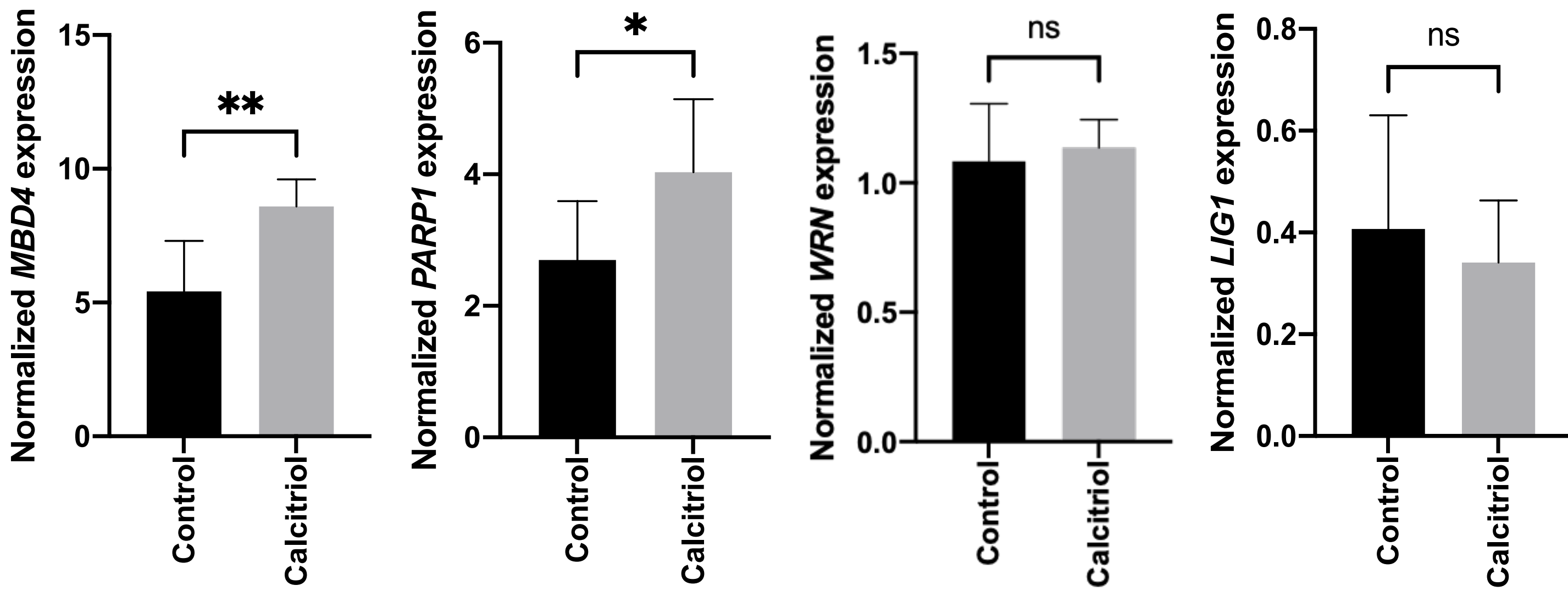

Supplement: Supplementary file 1 [file ijms-24-05488-s001.zip › Supplementary Figure S3.pdf]
